# Pharmacogenetic Associations with Statin Regimen Modification, Intolerance, and Adverse Outcomes in Coronary Artery Disease Patients

**DOI:** 10.3390/ph19030514

**Published:** 2026-03-21

**Authors:** Rania Abdel-latif, Shaban Mohammed, Mohamad Saad, Khalid Kunji, Wadha Al-Muftah, Ayman El-Menyar, Jassim Al Suwaidi

**Affiliations:** 1 Qatar Genome Program, Qatar Precision Health Institute, Qatar Foundation, Doha 5825, Qatar; rabdellatif@qf.org.qa (R.A.-l.); walmuftah@qf.org.qa (W.A.-M.); 2 Department of Pharmacology and Toxicology, Faculty of Pharmacy, Minia University, Minia 61511, Egypt; 3 Pharmacy Department, Hamad Medical Corporation, Doha 3050, Qatar; smohammed3@hamad.qa; 4 Qatar Computing Research Institute, Hamad Bin Khalifa University, Doha 5825, Qatar; msaad@hbku.edu.qa (M.S.); kkunji@hbku.edu.qa (K.K.); 5 Trauma and Vascular Research and Development, Hamad Medical Corporation, Doha 3050, Qatar; 6 Clinical Medicine Department, Weill Cornell Medical College, Doha 3050, Qatar; 7 Cardiology and Cardiovascular Surgery, Hamad Medical Corporation, Doha 3050, Qatar; jalsuwaidi@hamad.qa

**Keywords:** pharmacogenetic burden, statins intensity, statin intolerance, adverse event, myopathy, personalized therapy

## Abstract

**Background**: Statins are central to primary and secondary prevention of atherosclerotic cardiovascular disease but are often underutilized due to myopathy and intolerance. While individual pharmacogenetic (PGx) variants, particularly in *SLCO1B1*, are linked to statin-associated muscle symptoms, the real-world impact of both clinical and cumulative PGx burden on regimen modification and adverse outcomes remains unclear. We aimed to evaluate the existing uncertainty regarding whether combined PGx scores can effectively guide statin dose titration and regimen modification, thereby filling a key clinical gap. **Methods**: A retrospective cohort study of 911 statin-treated patients with coronary artery disease was conducted from the Qatar Cardiovascular Biorepository with available whole-genome sequencing data. Variants in *SLCO1B1*, *ABCG2*, and *CYP2C9* were combined into a functional PGx burden score, and their associations with statin regimen modification, intolerance, myopathy, liver injury, adherence, and composite adverse events were evaluated. The composite adverse events were defined as the occurrence of any statin-related adverse event, including statin-associated myopathy, liver injury, or poor medication adherence, during the follow-up period. Patients were classified as having experienced the composite outcome if at least one of these events occurred. **Results**: Over 12 months following statin initiation, 10.2% of patients underwent dose escalation, 11.4% de-escalation, and 78.4% remained on the same regimen. PGx burden is not statistically significantly associated with statin intolerance (OR 1.14; 95% CI: 0.73–1.76), composite adverse outcome (OR 1.08; 95% CI 0.82–1.42), or time to regimen change (HR 1.02; 95% CI 0.77–1.35). However, higher PGx burden showed a directional tendency toward dose de-escalation (RRR 1.18, 95% CI 0.76–1.84) and lower likelihood of escalation (RRR 0.93, 95% CI 0.56–1.54). **Conclusions**: Clinical factors, particularly statin intensity and myopathy, were the primary determinants of regimen modification. The PGx burden contributes to vulnerability to statin-related adverse effects in a context-dependent manner but does not independently drive statin regimen modification in routine clinical practice. Prospective studies are warranted to assess the clinical utility of PGx-guided workflows in statin therapy.

## 1. Introduction

Statins, inhibitors of 3-hydroxy-3-methylglutaryl-coenzyme A (HMG-CoA) reductase, are the cornerstone of lipid-lowering therapy and are widely prescribed for the primary and secondary prevention of atherosclerotic cardiovascular disease [1,2]. By inhibiting the rate-limiting step of hepatic cholesterol biosynthesis, statins effectively lower plasma low-density lipoprotein cholesterol (LDL-C) levels, resulting in substantial reductions in cardiovascular morbidity and mortality, as consistently demonstrated in large randomized clinical trials [3,4]. Despite their well-established efficacy and generally favorable safety profile, statin intolerance remains a significant clinical challenge. In routine clinical practice, up to 30–50% of patients discontinue or modify statin therapy within 1–2 years, most commonly due to statin-associated muscle symptoms (SAMS) [5,6,7]. SAMS is influenced by multiple factors, including drug–drug interactions, comorbidities, and genetic variability, leading to poor adherence and increased residual cardiovascular risk [8,9]. In 2020, the Clinical Pharmacogenetics Implementation Consortium (CPIC) guideline highlighted the role of multiple pharmacokinetic genes, including *SLCO1B1*, *ABCG2*, and *CYP2C9*, in guiding statin therapy, underscoring the extensive investigations into the contribution of genetic variability to interindividual susceptibility to SAMS [10]. The strongest and most consistent evidence relates to *SLCO1B1*, where the reduced-function c.521T>C (*5, p.V174A, rs4149056) variant impairs hepatic uptake via OATP1B1, leading to higher circulating statin concentrations and a well-established increase in myopathy risk, especially with simvastatin and, to a lesser extent, atorvastatin [11,12]. In addition, the c.388A>G variant (rs2306283) has been reported to modify *SLCO1B1* transport activity and, in combination with rs4149056, further influence the plasma concentration of the statins [13,14]. Consistent with this evidence, CPIC guidelines classify carriers of *SLCO1B1* decreased or poor function phenotypes, including the *5 and *15 haplotypes, as being at increased risk for SAMS. These guidelines recommend avoiding simvastatin and favoring lower doses or alternative statins, with consideration of creatine kinase monitoring when clinically indicated [10]. Variants in *ABCG2*, most notably c.421C>A (rs2231142), reduce efflux activity of the breast cancer resistance protein (BCRP), leading to increased systemic exposure to several statins, specifically atorvastatin and rosuvastatin [15,16]. *CYP2C9* reduced-function alleles (*2, *3) influence the metabolism of statins such as fluvastatin and contribute modestly to variability in statin exposure [17]. Evidence linking these variants to SAMS is limited, but CPIC incorporates *CYP2C9* genotype into dosing recommendations to minimize the risk of toxicity, particularly in the presence of interacting drugs [10]. The functional impact and clinical relevance of *SLCO1B1*, *ABCG2*, and *CYP2C9* variants in statin therapy are summarized in Table 1.

Given the multifactorial nature of SAMS, genetic predisposition alone, particularly when limited to a single *SLCO1B1* variant, is insufficient to explain clinical intolerance [18]. Beyond genetics, SAMS risk is influenced by concomitant medications, comorbid conditions, adherence behavior, and other real-world treatment factors [19]. Clinicians often manage statin-associated complications by transitioning patients from lipophilic agents to hydrophilic alternatives or to agents with a distinct metabolic profile. These swaps can minimize certain drug–drug interactions, but do not resolve the substantial interindividual variability in systemic exposure due to pharmacogenetic factors, along-side physiological and environmental determinants [20,21,22]. Although the pharmacokinetic effects of key statin pharmacogenetics are well established, most prior studies have focused on single-gene associations or statin-specific toxicity rather than cumulative genetic susceptibility within real-world prescribing contexts. Moreover, the clinical relevance of integrating multiple actionable variants with drug–drug interactions and patient-level characteristics remains incompletely defined [18]. Therefore, we investigated the combined impact of *SLCO1B1*, *ABCG2*, and *CYP2C9* genotypes, along with clinical and treatment factors, on statin intolerance and statin regimen modification in a large Middle Eastern cohort of patients with coronary artery disease (CAD) receiving statins for secondary prevention. This study aims to capture gene–drug–environment interactions and advance the translation of pharmacogenetics (PGx) into personalized statin therapy. As there is limited evidence, we sought to explore whether integrating multiple actionable PGx variants can meaningfully inform statin dose titration and regimen modification in routine clinical practice.

## 2. Results

### 2.1. Baseline Clinical and Treatment Characteristics

**Figure 1:**
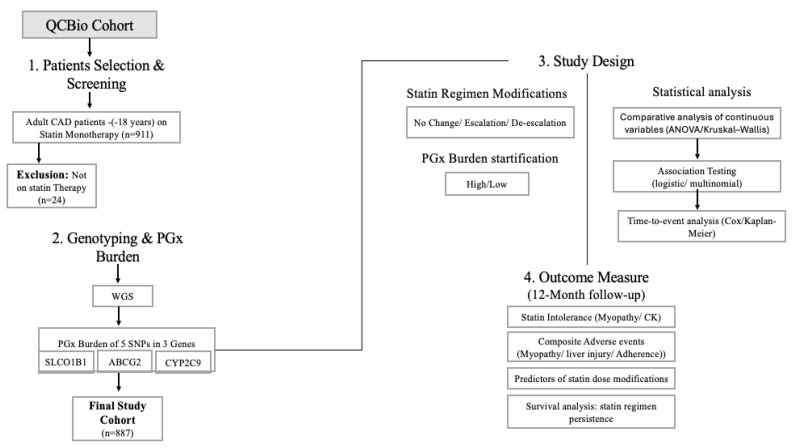
Study methodology and analytical workflow.

A total of 911 patients were included, of whom 10.21% underwent dose escalation, 11.42% underwent dose de-escalation, and 78.38% remained on the same statin drug and dose throughout a 12-month follow-up period. Figure 1 shows the study workflow.

Baseline demographic characteristics were largely comparable across groups, with no significant differences in age or smoking status. The proportion of males differed significantly, with the escalation group showing the lowest representation (60.22%, *p* = 0.025). Among comorbidities, asthma was most frequent in the escalation group (29.55%, *p* = 0.023), whereas diabetes, hypertension, chronic kidney disease, and heart failure showed no significant between-group differences.

For laboratory parameters, triglyceride levels demonstrated a statistically significant difference (*p* = 0.003), with higher levels observed particularly in the de-escalation group. In contrast, levels of cholesterol, high-density lipoprotein (HDL), low-density lipoprotein (LDL), creatinine, Alanine transaminase (ALT), and Aspartate transaminase (AST) were similar across groups.

Treatment-related characteristics showed the most apparent separation. Statin type distribution differed significantly (*p* = 0.001), with atorvastatin being most prescribed, but rosuvastatin was more frequent in the de-escalation group. Statin intensity also varied markedly (*p* = 0.001): the escalation group mostly initiated therapy at moderate intensity (79.7%), whereas the de-escalation group predominantly received high-intensity statins (75.8%). Concomitant use of enzyme inhibitors or inducers did not differ significantly between groups (Table 2).

### 2.2. Pharmacogenomic Profiles and Their Distribution Across Statin Regimen Change Groups

Genotype frequencies for *SLCO1B1* and *ABCG2* were broadly similar across the three-statin regimen change groups, with no statistically significant differences observed. In contrast, *CYP2C9* reduced-function alleles were significantly more prevalent in the escalation group, occurring 1.3 times more frequently than in the other groups. Specifically, the escalation group had a notable 43.01% proportion of reduced-function carriers (*p* = 0.006) (Table 3).

Phenotype-based classification of SLCO1B1 diplotypes showed no significant association with statin regimen change (*p* = 0.49). Normal-function phenotypes accounted for approximately two-thirds of each group, whereas decreased- and poor-function phenotypes were distributed similarly, with no meaningful deviation (Table 4).

**Figure 2:**
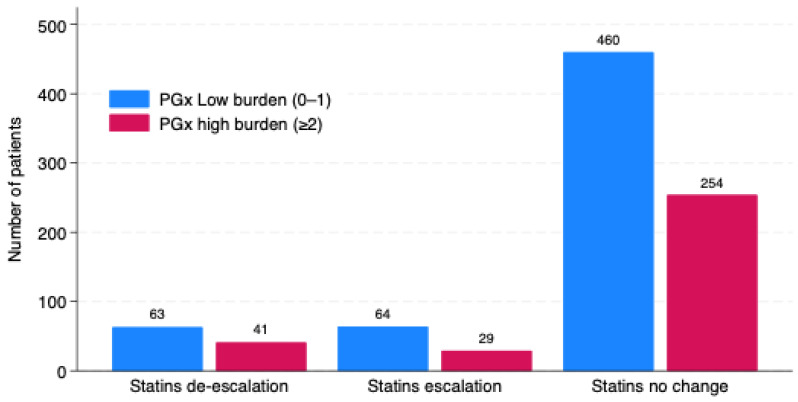
PGx burden across statin regimen change.

The distribution of the PGx burden score (0–4), representing the cumulative number of risk alleles across key statin-related genes, as shown in Figure 2, showed a predominance of low burden (scores 0–1) in all groups. A similar pattern across the escalation, de-escalation, and no-change groups was observed, without evidence of enrichment for higher PGx burden in any specific regimen-change category.

### 2.3. Clinical Outcomes and Muscle/Liver Biomarkers Across Statin Regimen Change Groups and Their Association with Pharmacogenomic Burden

Across statin regimen groups, rates of myopathy changed significantly (*p* = 0.027), with the highest rate observed in the de-escalation change group (23.08%). PGx burden categories showed no meaningful association with myopathy risk (all *p* > 0.44). Statin escalation was significantly associated (*p* = 0.002) with a higher frequency of liver injury, particularly among patients with low PGx burden. Poor adherence was most prevalent among patients whose statin regimen was not modified, particularly in those with low PGx burden. Table 5 shows that muscle and liver biomarkers did not differ significantly between statin regimen groups. Across all biomarkers, PGx burden categories showed no statistically significant associations.

### 2.4. PGx Burden and Composite Outcomes

As shown in Table 6, the composite adverse outcome occurred similarly in patients with low PGx burden (0–1) and those with high PGx burden (2–4). Composite events were observed in 40.7% of the low-burden group and 42.6% of the high-burden group. In the unadjusted analysis, patients with a high PGx burden showed a trend toward a higher risk of composite adverse outcomes; however, this did not reach statistical significance (OR = 1.08; 95% CI: 0.82–1.42; *p* = 0.582).

### 2.5. Multivariable Predictors of Statin Intolerance

**Figure 3:**
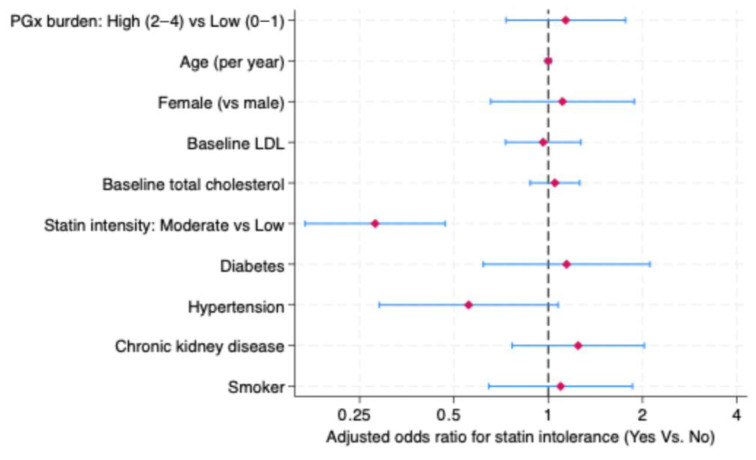
Adjusted associations between pharmacogenetic burden, clinical factors, and statin intolerance.

Patients with a high PGx burden showed a numerically higher risk of statin intolerance compared with those with a low burden. However, this association did not reach statistical significance (adjusted OR = 1.14; 95% CI: 0.73–1.76). Initiating a moderate-intensity statin was independently associated with lower odds of developing statin intolerance (OR = 0.28; 95% CI: 0.17–0.47). High-intensity statin therapy was omitted from the model due to collinearity. No significant associations were observed for age, gender, lipid parameters, cardiometabolic comorbidities, or smoking status after multivariate adjustment (Figure 3).

### 2.6. Predictors of Statin Dose Modification

**Figure 4:**
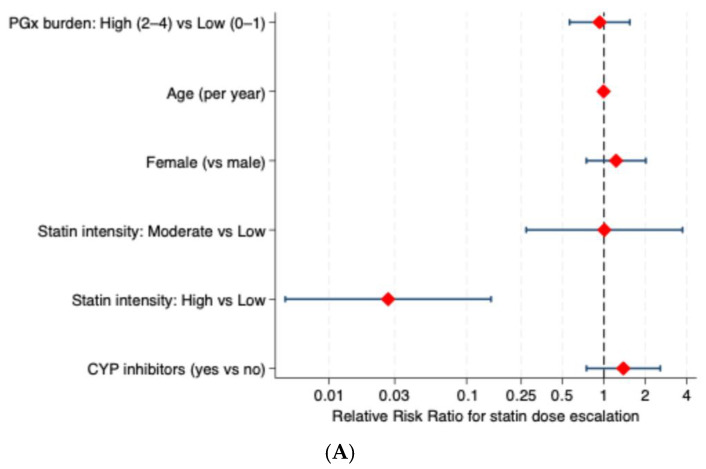

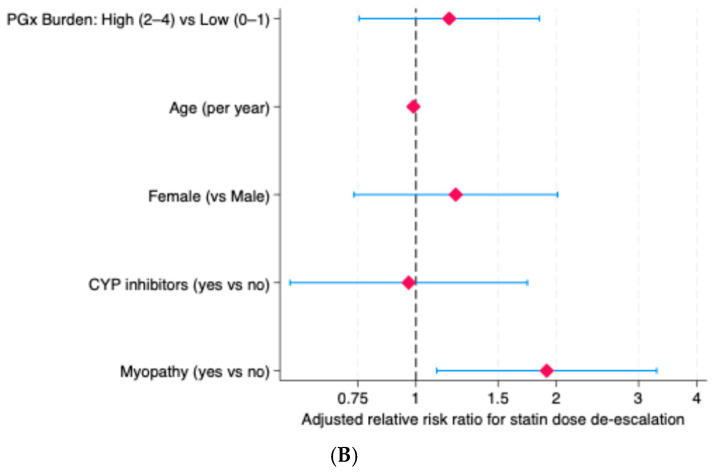
Predictors for statin dose regimen modifications. (**A**) Predictors for statin escalation with no change. (**B**) Predictors for statin de-escalation with no change.

PGx burden was not significantly associated with either statin dose escalation or de-escalation relative to no change. However, the direction of effect differed by outcome, with high PGx burden showing a tendency toward dose de-escalation (adjusted RRR = 1.18, 95% CI: 0.76–1.84) and a lower relative likelihood of dose escalation (adjusted RRR = 0.93, 95% CI: 0.56–1.54), although neither association reached statistical significance (Figure 4).

For escalation, Baseline statin intensity showed a strong differential effect. High-intensity statin therapy was associated with a substantially lower relative risk of subsequent dose escalation compared with low-intensity therapy (adjusted RRR = 0.03, 95% CI: 0.005–0.15), whereas moderate-intensity statin therapy was not significantly associated with escalation risk (adjusted RRR = 1.01, 95% CI: 0.27–3.73). Other demographic or clinical covariates did not independently influence escalation decisions in this cohort. Furthermore, Statin-associated myopathy was independently associated with an increased likelihood of dose de-escalation (adjusted RRR = 1.91, 95% CI: 1.11–3.28), indicating that clinical intolerance, rather than pharmacogenetic burden, primarily drove dose reduction decisions.

### 2.7. Association of Pharmacogenetic Burden with Time to Regimen Change

**Figure 5:**
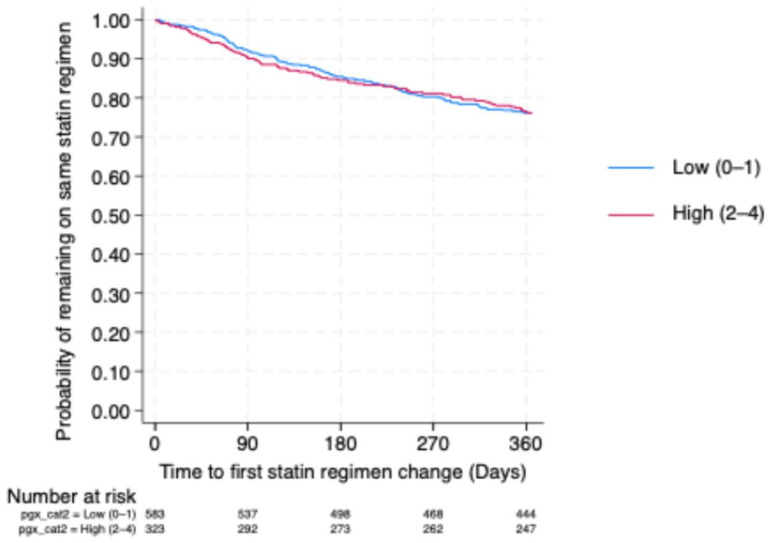
Kaplan–Meier estimates of remaining on the same statin regimen over 12 months stratified by PGx burden.

At 12 months, the estimated probability of remaining on the initial statin regimen was approximately 75–78% in both PGx burden categories. The survival curves for both groups largely overlapped throughout the follow-up period, with no evident early separation or divergence over time (Figure 5). Consistent with this, no significant association was observed between PGx burden and regimen persistence (HR 1.02, 95% CI: 0.77–1.35; *p* = 0.884).

## 3. Discussion

This study evaluated the influence of genetic and clinical factors on statin regimen modification and statin-associated adverse outcomes in a large real-world Middle Eastern CAD cohort. By focusing on well-characterized statin pharmacogenetics and functional alleles, alongside clinically relevant treatment variables, we sought to clarify how genetic susceptibility and routine prescribing practices interact to shape statin use, tolerability, and downstream outcomes in everyday clinical care. While CPIC recommendations are primarily statin- and variant-specific [10], a cumulative multi-gene approach may better reflect the multifactorial nature of statin disposition, particularly in real-world settings where multiple pathways influence exposure and tolerability.

To capture cumulative genetic susceptibility, we constructed a weighted functional PGx burden score that reflects the relative contributions of key statin disposition pathways. *SLCO1B1* was assigned a greater weight because impaired OATP1B1-mediated hepatic uptake represents the most consistently validated and clinically impactful mechanism underlying statin-associated muscle toxicity [10,12,23]. Although numerous *SLCO1B1* variants have been described, functional effects are largely driven by two common non-synonymous SNPs (c.388A>G and c.521T>C), with the latter defining the *5 and *15 haplotypes associated with reduced transporter function due to altered hepatic trafficking [23].

Notably, population-level analyses from the Qatar Genome Program have demonstrated that the minor allele frequency of *SLCO1B1* c.521T>C in the Qatari population is relatively high compared with several global subpopulations (up to 0.2322), further underscoring its clinical relevance in this cohort. In contrast, *ABCG2* c.421C>A exhibits lower frequency across most populations except East Asians. Additionally, CYP2C9*2 and *3 alleles also demonstrate relatively low and stable frequencies across ancestries [24]. Accordingly, *ABCG2* and *CYP2C9* were weighed more modestly, reflecting their statin-specific or pathway-limited effects on efflux transport and metabolic clearance. This pragmatic approach balances biological plausibility, clinical relevance, and statistical stability, yielding a composite score applicable across statin types in real-world practice while acknowledging heterogeneity in gene–statin interactions.

In this cohort, most patients remained on their initial statin regimen during follow-up, with dose escalation or de-escalation occurring in a minority, consistent with the overall stability of statin prescribing in routine care [25]. Baseline demographic and comorbidity profiles were largely comparable across modification groups, suggesting that regimen changes were not primarily driven by global cardiovascular risk or systemic disease burden. Instead, statin-related characteristics emerged as the dominant determinants of dose modification. Escalation was strongly associated with initiation of moderate-intensity therapy, consistent with guideline-directed stepwise titration toward lipid targets [26]. Conversely, de-escalation was predominantly linked to high-intensity statin use, supporting the interpretation that dose reduction frequently reflects tolerability concerns or biochemical changes rather than a lack of efficacy [27]. Our multinomial regression analyses further clarified these patterns. De-escalation was associated with myopathy, reinforcing the central role of muscle-related symptoms in prompting dose reduction, whereas escalation was driven primarily by statin intensity.

In the current cohort, none of the group studies showed high PGx burden mirroring the lack of genotype-driven dosing in practice. This finding further supports the argument that PGx burden does not distinctly influence regimen changes, underscoring the potential gap in applying genetic insights to clinical decision-making

Although high PGx burden was not statistically associated with dose escalation or de-escalation, patients with higher PGx burden showed a greater likelihood of de-escalation and a lower likelihood of escalation. Importantly, this pattern is consistent with real-world prescribing behavior, in which dose intensification may be avoided or reduced in response to early clinical or laboratory signals, rather than being guided by pre-emptive PGx information [28,29]. The lack of statistical significance likely reflects a combination of limited statistical power, heterogeneity in statin exposure, and the absence of preemptive PGx-guided prescribing, suggesting that genetic risk may influence clinical practice indirectly or in an attenuated manner in real-world settings. Previous studies showed that genotype may influence some statins prescribing changes but not uniformly [29]. In support, Ferri et al. emphasize that genetic predisposition alone is insufficient, and clinical factors must also be considered while considering the multifactorial nature of SAMS [18].

This interpretation is further supported by the time-to-event analysis, which demonstrated comparable 12-month persistence on the initial statin regimen across PGx burden categories, with no evidence of differential regimen switching. Together, these findings reinforce the notion that cumulative PGx risk alone does not strongly influence decisions to modify statin therapy once treatment has been initiated in routine clinical practice. PGx burden reflects probabilistic variation in statin disposition that may increase exposure without consistently reaching clinical thresholds that prompt regimen changes, particularly when moderate-intensity statins or conservative dose titration strategies are employed [30,31].

Evaluation of adverse outcomes, including myopathy, liver injury, and non-adherence, provided additional insight into the clinical impact of PGx burden. Although de-escalation of doses was more frequently associated with myopathy, the overall influence of PGx burden on the composite adverse outcome, statin intolerance and biomarker profile was modest, suggesting that genetic susceptibility alone is insufficient to drive clinical events in the absence of overt manifestations.

These findings align with prior large-scale observational studies. Analyses from the UK Biobank and other real-world cohorts have shown that *SLCO1B1* variants and myopathy exert largely independent effects, with no strong interaction detected, and that *SLCO1B1* variation does not uniformly predict statin-associated muscle symptoms or tolerability [32,33]. On the other hand, some statin-specific analyses have demonstrated clearer associations between reduced-function *SLCO1B1* alleles and intolerance, particularly for simvastatin; these effects appear to be exposure- and drug-dependent rather than class-wide [16,34,35]. In our stratified analyses of simvastatin and atorvastatin users, PGx burden was likewise not significantly associated with statin intolerance; however, consistently higher event rates suggest a directional tendency toward increased risk. Our findings are consistent with prior studies, underscoring that PGx is not a standalone predictor of adverse drug events but a complementary tool that, when integrated with clinical factors, helps identify at-risk patients and enables more precise, individualized therapy [36].

## 4. Materials and Methods

### 4.1. Study Population and Data Sources

Participants in this retrospective cohort study were recruited from the Qatar Cardiovascular Biorepository (QCBio) at Hamad Medical Corporation (HMC) [37], Doha, Qatar. This study involved patients with coronary artery disease from multiple sites, including the Cardiac Catheterization Laboratory, Coronary Care Unit, and Heart Hospital outpatient clinics. Participants underwent standardized phenotyping, clinical data collection, and biospecimen sampling were performed according to predefined biobank protocols. Genomic DNA was extracted using validated automated protocols and quantified prior to sequencing. Whole-genome sequencing was performed using high-throughput sequencing platforms. Variant calling, alignment to the reference genome, and quality-control filtering followed established pipelines as previously described [38].

The study protocol received approval from the Qatar Biobank Institutional Review Board (IRB Protocol #: E -2018-QF-QBB-RES-ACC-0077-0057). Written informed consent was obtained from all participants before enrollment in the QCBio cohort.

### 4.2. Statin User Identification

From the QCBio cohort, 911 statin users were identified. Statin use was defined as treatment with simvastatin, atorvastatin, pravastatin, rosuvastatin, or fluvastatin as monotherapy among QCBio participants.

### 4.3. Clinical and Medication Data Collection

Demographic characteristics, clinical diagnoses, laboratory measurements, and medication prescriptions were extracted from the electronic health records (EHR) of HMC. Detailed information on statin type, dose, treatment duration, and concomitant medications, with particular attention to drugs known to interact with statins, was obtained.

### 4.4. Assessment of Statin Exposure and Adherence

Pharmacy dispensing records were used to assess statin switching, discontinuation, and medication adherence. Adherence was estimated using the medication possession ratio (MPR), calculated as (MPR = cumulative medications dispensed within follow-up period/total days of follow-up). Patients with an MPR ≥ 80% were classified as adherent, whereas those with an MPR < 80% were classified as non-adherent, consistent with established definitions [39].

### 4.5. Definition of Statin Regimen Modification

Statin regimen changes were classified according to the American College of Cardiology (ACC) and the American Heart Association (AHA) statin intensity definitions [2]. Dose escalation was defined as a change from a lower- to a higher-intensity statin regimen, whereas dose de-escalation was defined as a change from a higher- to a lower-intensity regimen. No change was defined as continuation of the same statin type at the same dose, corresponding to unchanged statin intensity, throughout the follow-up period. Statin intensity categories and corresponding doses used for classification are summarized in Table 7.

### 4.6. Definition of Statin Intolerance Phenotypes

Biochemical evidence of statin intolerance was characterized by elevated creatine kinase (CK) levels, with sex-specific upper limits of normal of 120 IU/L for women and 180 IU/L for men [4,5]. Statin intolerance was further characterized by a 3-fold elevation of CK, accompanied by myopathy findings or statin dose deescalation or change in statin more than 3 times [40,41].

### 4.7. Definition of Myopathy and Composite Adverse Events

Recorded myopathy in EHR refers to disorders in which the primary pathology involves skeletal muscle, resulting in muscle weakness or dysfunction. Clinical phenotypes of myopathy were classified according to definitions adapted from ACC/AHA and the National Heart, Lung, and Blood Institute [42]. The spectrum of muscle-related adverse events included myalgia, myositis, rhabdomyolysis, asymptomatic myopathy, and statin-induced myopathy [7]. The composite adverse events were defined as the occurrence of any statin-related adverse event, including statin-associated myopathy, liver injury, or poor medication adherence, during the follow-up period. Patients were classified as having experienced the composite outcome if at least one of these events occurred.

### 4.8. PGx Burden Score

We constructed a PGx burden score from five loci *SLCO1B1* rs4149056 [12:21331549:T:C], *SLCO1B1* rs2306283 [12:21329738:A:G], *ABCG2* rs2231142 [4:89052323:G:T], CYP2C9*2 rs1799853 [10:96702047:C:T] and CYP2C9*3 rs4986893 [10:96540410:A:C] based on functional characterization and pharmacokinetic studies [10,20,43]. A PGx burden score was calculated based on the established functional patterns of the incorporated alleles. *SLCO1B1* was modeled using diplotype-derived transporter function phenotype, with normal function assigned 0 points, decreased function 1 point, and poor function 2 points. rs2231142 homozygosity was rare; therefore, *ABCG2* was modeled under a dominant framework where 0 was assigned for non-carriers, and 1 was assigned for any variant allele carrier to ensure stable estimation and avoid sparse-data bias. Similarly, *CYP2C9* reduced-function alleles (*2 or *3) were coded under a dominant model (0 = *1/*1; 1 = any reduced-function allele) to capture any reduction in enzymatic activity relevant to statin metabolism. The overall PGx burden score was calculated as the sum of these components. The weighted functional score was explored, assigning greater weight to *SLCO1B1* due to its more substantial and more consistent evidence for clinically relevant statin exposure and myopathy risk [10] (Table 8).

### 4.9. Statistical Analysis

Continuous variables were summarized as mean ± standard deviation (SD) or median (interquartile range; IQR), as appropriate, and compared across statin regimen-change groups using one-way ANOVA or the Kruskal–Wallis test. Categorical variables were summarized as counts and percentages and compared using the χ^2^ test. Associations between PGx variables and clinical outcomes were assessed using χ^2^ tests. Multivariable logistic regression models were constructed to assess the independent association between PGx burden and statin intolerance. Multinomial logistic regression was used to evaluate predictors of statin regimen modification. Multinomial and logistic regression analyses were adjusted for demographic and clinical covariates, including age, sex, baseline LDL-C levels, diabetes status, baseline statin intensity, and concomitant medications, to account for potential confounding effects. Time to first statin regimen change within 12 months was analyzed using Cox proportional hazards models, with Kaplan–Meier curves for visualization and the log-rank test for comparison. Data were expressed as odds ratio (OR), relative risk ratio (RRR), Hazard ratio (HR), and 95% confidence interval (CI); whenever applicable. Analyses were performed using Stata version 18.5 (StataCorp, College Station, TX, USA), with *p* < 0.05 considered statistically significant.

Study limitation: The retrospective nature of this study and single-center data are limitations that cannot rule out the selection bias and unmeasured confounders. This study was conducted in a real-world CAD population with substantial comorbidity and polypharmacy, which may have attenuated the independent effects of PGx burden despite multivariable adjustment. Although the performed analyses were based on prespecified hypotheses, we acknowledge that multiple statistical comparisons were performed, and findings should be interpreted cautiously within the context of the overall study design and the specific population examined. The multiple comparisons could generate a type I error, and the findings should be interpreted cautiously. Furthermore, statin intolerance and myopathy were identified from EHR data and laboratory thresholds, which may not fully capture patient-reported muscle symptoms. It is worth noting that the cohort was predominantly treated with atorvastatin and rosuvastatin, limiting statin-specific analyses, particularly for simvastatin, where PGx effects are most pronounced. In addition, PGx testing was not implemented preemptively, and prescribing decisions were made without access to genetic information, likely reducing the observable clinical impact of PGx burden.

## 5. Conclusions

The study findings suggest that PGx burden may shape the direction of treatment modification, favoring de-escalation over escalation rather than the overall frequency of regimen changes. The modest effect observed in this retrospective, non-genotype-guided cohort likely reflects routine clinical practice where dosing decisions were not informed by PGx results. The clinical impact of PGx information is therefore likely to be maximized when integrated into structured PGx-guided workflows, combined with standardized intolerance phenotyping, adherence assessment, and exposure-matched dosing strategies. Prospective studies incorporating pre-emptive PGx testing and clinical decision support are needed to clarify the utility of PGx burden in optimizing statin therapy and reducing adverse outcomes in routine practice.

## Figures and Tables

**Table 1:** Key pharmacogenetic variants relevant to statin therapy.

Gene	Variant (rsID)	Encoded Protein	Functional Consequence	Mechanistic Effect	Clinical Implication	CPIC Consideration
*SLCO1B1*	c.521T>C (rs4149056)	OATP1B1 (hepatic uptake transporter)	Reduced OATP1B1 transporter function	Decreased hepatic uptake → Increased systemic statin exposure	Increased risk of statin-associated muscle symptoms (SAMS), especially with simvastatin	Avoid simvastatin; consider lower dose or alternative statin; CK monitoring if indicated
	c.388A>G (rs2306283)	OATP1B1	Altered transporter activity	Modifies *SLCO1B1* function (substrate-dependent)	May influence plasma statin concentration	Incorporated into diplotype-based phenotype
*ABCG2*	c.421C>A (rs2231142)	BCRP (efflux transporter)	Reduced BCRP efflux function	Increased systemic exposure to atorvastatin and rosuvastatin	Potential increased myopathy risk (statin-specific)	Dose consideration for certain statins
*CYP2C9*	*2 (rs1799853)	CYP2C9 enzyme	Reduced enzymatic activity	Decreased metabolic clearance (fluvastatin)	Modest increase in statin exposure	Dose consideration for certain statins
	*3 (rs4986893)	CYP2C9 enzyme	Reduced enzymatic activity	Decreased metabolic clearance	Increased drug levels (substrate-dependent)	Dose consideration for certain statins

**Table 2:** Baseline characteristics by outcome group.

Variable	Overall	Escalation	De-Escalation	No Change	*p*-Value
N	911	93 (10.21%)	104 (11.42%)	714 (78.38%)	
**Demographics**					
Age (years, mean ± SD)	60.33 ± 10.71	61.71 ± 9.47	58.37 ± 10.32	60.43 ± 10.88	0.09
Male (%)	657 (72.12%)	56 (60.22%)	75 (72.12%)	526 (73.67%)	0.025
Smoker	222 (25.49%)	22 (25.00%)	29 (29.59%)	171 (24.96%)	0.613
**Comorbidity**					
Diabetes	715 (82.09%)	79 (89.77%)	81 (82.56%)	555 (81.02)	0.13
Hypertension	756 (86.80%)	92 (93.18%)	81 (82.65%)	593 (86.57%)	0.099
Chronic kidney diseases	268 (30.77%)	26 (29.55%)	34 (34.69%)	208 (30.36%)	0.663
Heart Failure	265 (30.42)	27 (30.68%)	36 (36.73%)	202 (29.49%)	0.345
Asthma	175 (20.09)	26 (29.55%)	24 (24.29%)	125 (18.25%)	0.023
peripheral artery disease	69 (7.92)	9 (10.32)	10 (10.20%)	50 (7.30)	0.426
Hypothyroidism	147 (16.88%)	16 (18.18)	22 (22.45)	109 (15.91)	0.255
Stroke	61 (7.0)	6 (6.82)	10 (1–0.20)	45 (6.57%)	0.418
**Baseline laboratory tests**					
Total cholesterol (mmol/L)	5(4.2–6)	5.37 (4.4–6)	5 (4.2–6.1)	5 (4.1–5.96)	0.219
LDL (mmol/L)	2.48 ± 0.99	2.59 ± 0.89	2.54 ± 1.09	2.45 ± 0.98	0.36
HDL (mmol/L)	1.03 (0.87–1.2)	1.07 (0.91–1.23)	0.97 (0.84–1.2)	1.03 (0.88–1.2)	0.09
TGs (mmol/L)	1.5 (1.1–2)	1.6 (1.2–2.03)	1.74 (1.3–2.3)	1.47 (1.1–2)1.09	0.003
Creatinine (µmol/L)	80 (68–99.7)	78.9 (62–97.3)	78 (65.5–100)77.99	80.1 (68.6–99.7)	0.43
ALT (U/L)	19 (13–26)	18 (13–23)	19 (13–26)	19 (13–27)	0.267
AST (U/L)	17 (14–22)	17 (13–22)	18(14–23)	17(14–22)	0.567
**Statin type**					0.244
Atorvastatin	559 (61.36)	48 (51.61)	65.38 (65.38)	443 (62.04)	
Rosuvastatin	307 (33.70)	40 (43.01%)	32 (30.77)	235 (32.91)	
Pravastatin	22 (2.41)	1 (1.08)	1 (0.96)	20 (2.80)	
Simvastatin	20 (2.20)	4 (4.30)	2 (1.92)	14 (1.96)	
Fluvastatin	3 (0.33)	0 (0)	1 (0.96)	2 (0.28)	
**Statin Intensity**					0.001
Low	15 (1.56)	3 (3.23)	0 (0)	12 (1.68)	
Moderate	406 (44.57)	87 (93.55)	18 (17.31)	301 (42.16)	
High	490 (53.79)	3 (3.23)	86 (82.69)	401 (56.16)	
**Interacting medications**					
Any inhibitor %	153 (16.79)	17 (18.28)	18 (17.31)	118 (16.53)	0.903
Any inducer %	3 (0.33)	1 (1.08)	1 (0.96)	1 (0.14)	0.163

Enzyme inhibitors: diltiazem, verapamil, azoles antifungals, cyclosporine, amlodipine. Enzyme inducers: carbamazepine, phenytoin, rifampin. Classification based on known effects on statin metabolism and transport pathways.

**Table 3:** Genotype distribution across statin regimen change groups.

Genotype/PGx Marker	Genotype	Escalation	De-Escalation	No Change	*p*-Value
*SLCO1B1* c.521T>C	TT	69.89%	60.58%	64.29%	0.692
	TC	27.96%	32.69%	30.25%	
	CC	1.08%	5.77%	4.48%	
*SLCO1B1* c.388A>G	AA	23.66%	27.88%	26.61%	0.139
	AG	55.84%	38.46%	46.87%	
	GG	18.28%	32.69%	25.07%	
*ABCG2* c.421G>T	GG	89.25%	86.54	87.82%	0.631
	GT	9.68%	11.54	11.62%	
	TT	1.08%	0.96	0.42%	
*CYP2C9* *2/*3 carriers		28.01% carriers	43.01% carriers	28.01% carriers	0.006

**Table 4:** Distribution of *SLCO1B1* phenotypes across statin regimen change groups.

*SLCO1B1* Diplotype	*SCLO1B1* Phenotyping	Total	Escalation	De-Escalation	No Change	*p*-Value
*1/*1, *1/*37, *37/*37	Normal Function	578 (65.02)	62 (69.66%)	63 (61.76%)	453 (64.90%)	0.49 for all
*1/*5, *1/*15, *37/*5, *37/*15	Decrease function	272 (30.60)	26 (29.21%)	33 (32.35%)	213 (30.60%)	
*5/*5, *5/*15, *15/*15	Poor function	39 (4.39)	1 (1.12)	6 (5.88)	32 (4.58)	

**Table 5:** Clinical outcomes and muscle/liver biomarkers across statin regimen change groups and their association with pharmacogenomic burden.

Outcome/Biomarkers	Escalation	De-Escalation	No Change	*p* (Group Comparison)	*p* (Group Comparison)	PGx Burden Association (*p*-Value)
**Outcomes**						
Myopathy (%)	14 (15.05%)	24 (23.08%)	94 (13.17%)	0.027	0.027	Low (0–1): 0.057 High (2–4): 0.234
Liver injury (%)	7 (7.53%)	4 (3.85%)	12 (1.68%)	0.002	0.002	Low (0–1): 0.026 High (2–4): 0.059
Non-adherence < 80% (%)	14 (15.05%)	17 (16.35%)	238 (33.33%)	<0.001	<0.001	Low (0–1): 0.001 High (2–4): 0.113
**Biomarkers**						
CK (U/L) (Median, Q1–Q3)	679.50 (342.75–1454.75)	320.50 (231.75–440.25)	295.00 (247.00–491.50)	0.074	Low (0–1): 0.105; High (2–4): 0.641	Low (0–1): 0.105; High (2–4): 0.641
Myoglobin (ng/mL) (Median, Q1–Q3)	261.00 (142.00–2521.00)	1964.00 (109.00–3819.00)	204.00 (149.00–423.50)	0.913	Low (0–1): 0.781; High (2–4): 1.000	Low (0–1): 0.781; High (2–4): 1.000
Troponin (ng/mL) (Median, Q1–Q3)	463.30 (179.80–2469.25)	303.50 (76.40–885.00)	301.20 (90.60–1032.00)	0.405	Low (0–1): 0.876; High (2–4): 0.168	Low (0–1): 0.876; High (2–4): 0.168
TSH (mIU/L) (Median, Q1–Q3)	1.98 (1.23–2.94)	1.96 (1.17–2.77)	1.97 (1.25–2.93)	0.910	Low (0–1): 0.465; High (2–4): 0.093	Low (0–1): 0.465; High (2–4): 0.093
Vitamin D (ng/mL) (Median, Q1–Q3)	19.00 (15.00–27.50)	21.00 (15.00–27.00)	19.00 (14.00–26.00)	0.560	Low (0–1): 0.895; High (2–4): 0.259	Low (0–1): 0.895; High (2–4): 0.259
Creatinine (µmol/L) (Median, Q1–Q3)	87.00 (69.25–109.75)	91.00 (72.00–118.00)	88.00 (73.00–114.00)	0.598	Low (0–1): 0.794; High (2–4): 0.646	Low (0–1): 0.794; High (2–4): 0.646

**Table 6:** Association between pharmacogenetic burden categories and composite adverse outcomes.

PGx Burden Category	Composite Outcome: No (%)	Composite Outcome: Yes (%)	OR	95% CI	*p*-Value
Low (0–1) (ref)	348 (59.28%)	239 (40.72%)	1.0	Reference	
High (2–4)	187 (57.41%)	138 (42.59%)	1.08	0.82–1.42	0.582

**Table 7:** Statin intensity categories and dose ranges used to classify statin regimen modification.

Statin	Low Intensity Dosage (mg)	Moderate Intensity Dosage (mg)	High Intensity Dosage (mg)
Atorvastatin	-	10–20	40–80
Rosuvastatin	-	5–10	20–40
Simvastatin	10	20–40	-
Pravastatin	10–20	40–80	-
Fluvastatin	20–40	80	-

Low intensity: LDL-C reduction < 30%; moderate intensity: LDL-C reduction 30–49%; high intensity: LDL-C reduction ≥ 50%.

**Table 8:** Definition of the PGx burden score.

Gene	Variant (rsID)	Functional Effect	Genetic Model	Coding Scheme	Maximum Score
*SLCO1B1*	Diplotype-based phenotype (rs4149056 c.521T>C + rs2306283 and c.388A>G) +	Reduced transporter activity	Phenotype-based	0 = Normal function (*1/*1, *1/*37, *37/*37) 2 = Decreased or Poor function (*1/*5, *1/*15, *37/*5, *37/*15, *5/*5, *5/*15, *15/*15)	2
*ABCG2*	c.421G>T (rs2231142)	Reduced efflux capacity (BCRP)	Dominant	1 = GT/TT, 0 = GG	1
*CYP2C9*	*2 or *3	Reduced enzymatic activity	Dominant	1 = any *2/*3, 0 = *1/*1	1
Total PGx Burden Score	—	Cumulative functional impairment in statin disposition	—	Sum of all components	0–4

## Data Availability

The datasets generated and/or analyzed during the current study are available from the corresponding author on reasonable request and signed data sharing agreement with the Medical Research Center, Hamad Medical Corporation, Doha, Qatar.

## Abbreviations

ACC	American College of Cardiology
AHA	American Heart Association
ALT	Alanine aminotransferase
AST	Aspartate aminotransferase
BCRP	Breast Cancer Resistance Protein
CAD	Coronary artery disease
CI	Confidence interval
CK	Creatine kinase
CPIC	Clinical Pharmacogenetics Implementation Consortium
EHR	Electronic health record
HDL	High-density lipoprotein
HMC	Hamad Medical Corporation
HR	Hazard ratio
HMG-CoA	3-hydroxy-3-methylglutaryl-coenzyme A
LDL	Low-density lipoprotein
LDL-C	Low-density lipoprotein cholesterol
MAF	Minor allele frequency
MPR	Medication possession ratio
OR	Odds ratio
PGx	Pharmacogenetics
QCBio	Qatar Cardiovascular Biorepository
RRR	Relative risk ratio
SAMS	Statin-associated muscle symptoms
